# Delusion or obsession: Clinical dilemma

**DOI:** 10.1186/1756-0500-5-384

**Published:** 2012-07-28

**Authors:** Priya Gandhi, Amin Muhammad Gadit

**Affiliations:** 1 Resident, Discipline of Psychiatry, Memorial University of Newfoundland, Newfoundland, Canada; 2 Professor of Psychiatry, Memorial University of Newfoundland, Newfoundland, Canada

**Keywords:** Delusion, obsession, psychosis, phenomenology

## Abstract

**Background:**

A 52-year old lady presented for admission with severe depression characterised by suicidal ideation and delusional belief.

**Case presentation:**

Her treatment regime was reviewed and modified. The dilemma was whether she suffered from a psychotic depression with delusion or an obsessional disorder. She responded well to change of antipsychotic medication.

**Conclusions:**

Her depression went in remission and her delusional belief decreased in intensity. She also gained reasonable insight into her problem. She is currently being followed up in the psychiatric outpatient clinic.

## Background

The academic difference between obsession and delusion is obvious. The obsessions often relate to ideas around contamination, symmetry and/or aggressive impulses but are recognized as irrational and product of own mind by the patients whereas delusions are firm fixed, false beliefs that are held true despite very strong evidence to suggest that these are incorrect. In a study [1], conclusions were made that “OCD and psychosis may coexist and may be unrelated. An obsession may become a delusion or vice versa and obsessions may trigger a psychotic episode. Obsessions may be misdiagnosed as delusions or hallucinations, recognition and treatment of OCD may improve the outcome of psychosis”.

## Case presentation

A 52 year old lady was referred for admission because of increasing severity in depressive symptoms. She had fleeting suicidal thoughts and expressed a belief that a former co-patient was sitting on her brain interfering with her daily activities. She reported a turbulent life history. She was born and raised in a remote community; her childhood remained uneventful and carefree. She completed high school and then took some courses in a trade school. After completing the courses, she started working as a health care support worker for few years, took a break and then resumed after she got married in 1985. Her spouse was quite abusive and subjected her to domestic violence. She remained extremely unhappy with her marital life. She finally got separated from her husband. She has two sons, ages 25 and 20 who live in a different area. Her younger son met an accident and lost his eye sight but is functional and working. The accident and subsequent events were quite stressful for her. She stopped working in the mid of 2011 as her depression and suicidal ideation became disabling. She did not report any substance abuse, legal issues or allergies but had ‘high cholesterol’ problem. She was admitted three times during the year 2011 before this admission. In terms of family history, her mother had committed suicide and sister suffered from depression. She herself had previous history of suicidal attempt. Her current admission was also precipitated following an overdose. Her depression accompanied by delusion of being overpowered was not responding to the previous treatment regime. Upon referral, there was a suggestion for a trial of Electroconvulsive Therapy (ECT). Following admission, her mental status examination revealed severe depression with fleeting suicidal thoughts, delusion of a former male co-patient overpowering her brain with limited insight into her problem. She was initially given a diagnosis of Major depressive disorder with psychotic features.

This diagnosis was based on DSM IV criteria for major depressive disorder. All her blood work and MRI were normal in findings. By definition in terms of phenomenology, delusion is a belief, held with extraordinary conviction, unshakeable, with incomparable subjective certainty and in contrast to person’s socio-cultural background. The patient in this case report stated that a person is overpowering her brain “sitting on my brain”, expressed firm belief of its reality and was unshakeable despite arguments presented against this belief. She was on Mirtazapine45 mg, Clonazepam 0.5 mg twice per day and Quetiapine800 mg per day without any beneficial effect before being referred for admission. Her treatment was reviewed by the multidisciplinary team. The dosage of Mirtazapine was maximised to 60 mg HS, Quetiapine was discontinued and Risperidone was introduced at 1 mg dose. Over the next two weeks, the dosage of Risperidone was titrated up to 4 mg. The patient showed very good response to this combination, her mood improved tremendously, delusions became shaky, gained adequate insight into her problem and dismissed the brain-occupation by the fellow patient as absurd and obsessional. She stated that “The occupation of my brain could be just my thought, it may not be real but it is quite undesirable”. This falls well into the quality of an obsession which is troubling thought, being recognized as once own, cannot get rid of a content of consciousness, realization of it being senseless and persisting without cause. The consideration for ECT was dropped, she was given off unit privileges and overnight passes. A family meeting was arranged in which it was expressed by the family members that she was doing quite well and they were pleased with the improvement.

The current case, presented with major depression with the firm belief that her brain was possessed by a man. She was considered to be suffering from Major depression with psychosis. With change of antipsychotic medication, the intensity of symptoms became less severe; she gained insight into her problem and started to believe that her thought was irrational. The journey appeared to be from delusion to obsession. This is in light with the above conclusions made in the study [1]. The same author had examined seven cases with features of OCD with psychosis and six of those responded well to addition of antipsychotic or antidepressants with good effect. ECT treatment was deferred in the current case in order to observe the symptoms and effect of prescribed medications. There was no strong evidence available for efficacy of ECT in obsessional disorder. However, one uncontrolled case series reported considerable improvement in such cases year after the treatment although improvement was correlated with improved depression scores [2]. In the current case, the patient was observed for improvement on the changed antipsychotic medication. A study [3] describes co-morbid OCD with schizophrenia. In this, they mention that OCD was associated with more severe depressive symptoms, social dysfunction and worse premorbid functioning. There remains a significant degree of uncertainty and complexity surrounding the issue of distinguishing between delusion and obsessive thought. [4]

Some authours feel that these symptoms are distinct entities and are distinguishable on the basis of the level of insight a patient displays. [5]

Apparently, this is a simple case but the intriguing factor was related to the phenomenology. Initially she remained firm about her belief giving a clinical impression of ‘delusion’ fulfilling all criteria of the same. With initiation of changed treatment she gave the picture of an obsession. With multiple interviews, it remained inconclusive whether the initial picture was of delusion or obsession. The case came under debate based on the issue of phenomenology whether the interview approach was inappropriate or the understanding of phenomenology was inadequate. Addition or change of antipsychotic medication can address both issues making the matter more complex. For training residents and clerks, the terms for psychopathology become more pertinent when it is clinically applied. In major depression with psychotic features or depression that is refractory to antidepressant medication, addition of antipsychotics may prove effective. This has been demonstrated in studies. [6]

In a study, it was concluded that antidepressant-antipsychotic cotreatment was superior to monotherapy with either drug class in the acute treatment of psychotic depression. [7]

The medical literature does not clearly mention the need for antidepressants in presence of pure psychotic features. In Obsessive-Compulsive Disorder (OCD), Quetiapine augmentation to SSRI’s was suggested to be beneficial in patients with treatment resistant OCD. [8] Hence, it is obvious that though (selective serotonin reuptake inhibitor)SSRI is the medication of choice in OCD, addition of an antipsychotic may prove beneficial.

A lesson was however learnt that addition/change of antipsychotic medication may affect mood related disorder, obsession and/or psychosis. This dilemma prompted us to write a case report for the general interest of readers. Though such type of presentations are frequently reported in academic literature, it gives repeated updates about this sort of clinical dilemmas and provide insight into the management issues.

## Conclusions

In terms of phenomenology, it was apparent that obsession and delusion can at times become difficult to differentiate. Literature based evidence demonstrates that combination of antidepressant and antipsychotic medication may benefit depression, with psychosis. Similarly, in resistant OCD, addition of atypical antipsychotic medication helps in bringing improvement in clinical profile. In this case report, there was a marked improvement in mood, intensity of delusional belief was weakened and insight was regained. Finally, she was discharged with a follow-up appointment in two weeks.

## Consent

"Written informed consent was obtained from the patient for publication of this case report and any accompanying images. A copy of the written consent is available for review by the Editor-in-Chief of this journal."

## Competing interests

The authors declare that they have no competing interests.

## Authors’ contributions

Both authors have made equal contribution in preparing this case report.
